# The coexistence of obesity and hunger in Latin America and the Caribbean

**DOI:** 10.1016/j.lana.2023.100653

**Published:** 2023-12-15

**Authors:** The Lancet Regional Health – Americas

Overweight and obesity are growing issues in Latin American and Caribbean (LAC) countries, threatening the health and wellbeing of millions. Overweight in children younger than 5 years is increasing at alarming rates, and a quarter of the adult population lives with obesity. Approximately 43.2 million people suffer from hunger in LAC countries and food insecurity affects 37.5% of Latin Americans and Caribbeans, according to the 2023 UN Regional Overview of Food Security and Nutrition. The concomitant urgency to fight obesity and hunger might seem paradoxical, but their roots are not that different: economic inequality, extensive monoculture focused on high-value products like soybeans and wheat, unfair trade practices that hurt smaller farmers, and poverty make healthy food inaccessible for most.

Data from the 2023 UN report shows that rates of obesity and overweight in the region tripled in the last 50 years and are significantly higher than the global average. In 2016, obesity prevalence in adults in LAC was double the global average (24.2% *vs* 13.1%) and it has continued to grow. This scenario is seen as a consequence of the rapid economic development in many LAC countries over the past decades that was accompanied by lifestyle changes with direct implications for people's health. A more Westernised diet and increased access to ultra-processed foods, paired with disorganised rural–urban migration and increased physical inactivity helped increase overweight and obesity.

Although often described as a recent event, the transformation of eating habits in LAC, often referenced as nutritional transition, seems to have started as early as in the 19th century, and was heavily influenced by the food and beverage industry and its dominance of the LAC regional market. A historiographic study looking back as far as 1850 describes the long history of the pathologisation of indigenous and local food, which were claimed to be poor in nutrients so that food companies could introduce their products in the local market. The study suggests that the nutritional transition started much earlier and was overlooked for many decades for reasons including the absence of data to cultural attitudes about weight and its relationship with wealth and health; thus, the current worrisome rates are a result of a very slow incremental process and not a sudden epidemic. Regardless of when they started and how fast they grew, overweight and obesity reached dangerous prevalence rates in the region that warrant immediate action.

With strong lobbying and production capacity, food companies can sell ultra-processed foods at low market prices compared to local farmers; and the appealing marketing of products as easier and “packed with essential vitamins”, can influence parents—especially those with lower education and reduced resources—to opt for the cheaper, less nutritional option. Several public health measures were put in place in LAC countries to reduce the consumption of unhealthy products. A growing list of countries have implemented mandatory front-of-pack warnings on products with high fat and sugar content. Barbados and Mexico implemented taxes on sugary drinks, and in November, Colombia became the first country in the region to implement a tax on all ultra-processed food. The idea behind those taxes is to reduce the consumption of said food and, albeit successful, for a greater impact, taxation of unhealthy products must be paired with actions that guarantee access to the healthy option. According to Panorama 2023, LAC has the highest cost for a healthy diet in the world, and this cost continues to grow, particularly in the Caribbean. Currently, one in five people in the region cannot access a healthy diet.

The reduced access to healthy foods is reflected not only in poor diet choices but also in no choices at all. Food insecurity, defined as having to reduce the quality or quantity of food consumed or going hungry, affected 247.8 million people in LAC in 2022. In the Caribbean, 60.6% of the population experienced moderate or severe food insecurity that year. Different groups of the population are unequally affected, with women and residents of rural and peri-urban areas being the most vulnerable. Moderate to severe food insecurity in rural areas was 8.3% higher than in urban centres in 2022. The current scenario of climate emergency is a direct threat to food production systems and preventive measures are imperative to increase the resilience of our food systems while prioritising the most vulnerable populations, who are systematically left behind. An urgent reformulation of food systems should focus on subsidising agriculture diversification, incentivising the production of more nutritional crops (like cassava), protecting fair trade, and improving distribution, with public policies that promote and secure affordable diets for all.

Unacceptably, 6.5% of the LAC population still suffers from hunger. The figures have slightly improved in 2022 but are still higher than the pre-COVID-19 levels and moving further away from meeting the SDG-2 of Ending Hunger by 2030. In a recent interview, Prof Jonathan D Ablard (Ithaca College, NY, USA) asserts that, “Both [hunger and obesity] are indicators of social and economic inequality, racial disparities in health, conflicts of interest between state actors and business, and a general abandonment of the state in its responsibility to care for the most vulnerable.” Ending hunger and improving malnutrition is a single fight against inequalities, and the fighters must define their strategies by putting the most vulnerable ones in the centre, aiming to achieve healthy eating for all. Luckily, this can be a fight with multiple winners.

